# Supplementary material for: Structural basis of ECF-σ-factor-dependent transcription initiation
Source: Nat Commun. 2019 Feb 12;10:710. doi: 10.1038/s41467-019-08443-3 (PMC6372665; doi:10.1038/s41467-019-08443-3)
Supplement: Supplementary file 1 — Supplementary Information [file 41467_2019_8443_MOESM1_ESM.pdf]

Structural basis of ECF-σ-factor-dependent transcription initiation

Wei Lin, Sukhendu Mandal, David Degen, Min Sung Cho, Yu Feng, Kalyan Das, and Richard H. Ebright

Supplementary Information

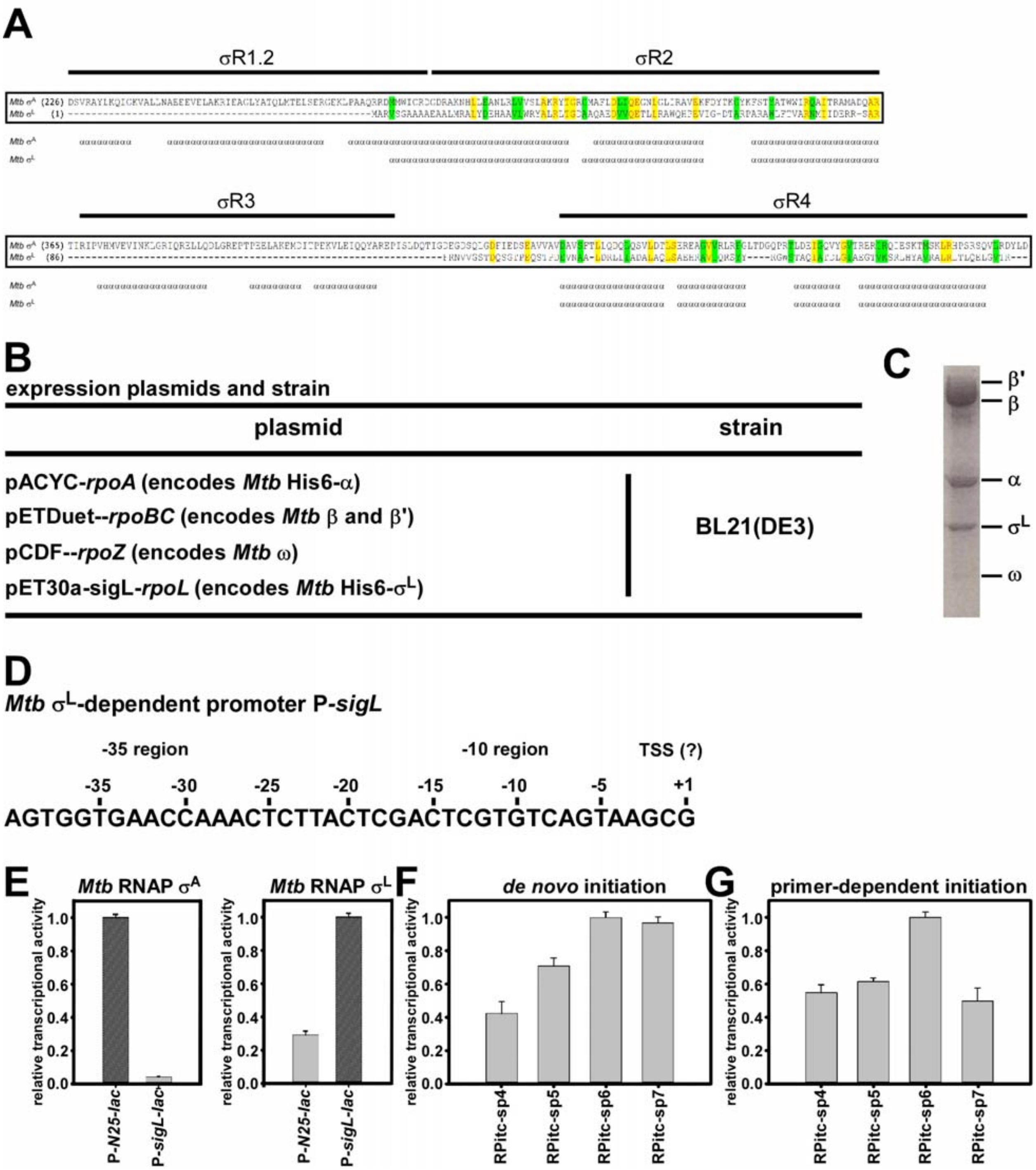

Supplementary Figure 1 (related to Fig. 1). Structure determination: *Mtb* RNAP-σ^L holoenzyme and *Mtb* σ^L-dependent promoter P-*sigL*

(A) Sequence alignment of *Mtb* σ^A (residues 1 to 225 omitted) and *Mtb* σ^L. σ conserved regions (σR1.1, σR1.2, σR2, σR3, and σR4) are shown above sequences. Helices, defined from crystal structures in Lin et al., 2017 and in this work (Figs. 1-2), are shown below sequences.

(B) Plasmids and strain used for production of *Mtb* RNAP-σ^L holoenzyme in *E. coli*.

(C) Coomassie-stained SDS-polyacrylamide gel electrophoresis of *Mtb* RNAP-σ^L holoenzyme produced in *E. coli*. Staining levels of β', β, α, ω, and σ^L indicate β':β:α:ω:σ^L stoichiometry is 1:1:2:1:1. [*Mtb* ω stains weakly (*1*).]
(D) Sequence of *Mtb* σ^L-dependent promoter P-*sigL* showing -35 region, -10 region, and reported transcription start site (TSS; *2-3*; see, however, *4*).
(E) Transcription experiments demonstrating *Mtb* RNAP σ^A holoenzyme selectively recognizes σ^A-dependent promoter P-N25, and *Mtb* RNAP σ^L holoenzyme selectively recognizes σ^L-dependent promoter P-*sigL*. The magnitude of selectivity of *Mtb* RNAP σ^L holoenzyme for the σ^L-dependent promoter P-*sigL* (3-fold) is in the range observed for the ten *Mtb* RNAP ECF σ factors (2- to 30-fold). Error bars, SE (N = 3).
(F) Transcription experiments demonstrating *de novo* transcription initiation by *Mtb* RNAP-σ^L holoenzyme on P-*sigL* derivatives having spacer ("sp") lengths of 4, 5, 6 and 7 bp (sequences in Supplementary Fig. 2; transcription start at position +1 in each case at template-strand position 2 nt upstream of nucleic-acid-scaffold dsDNA segment). Error bars, SE (N = 3).
(G) Transcription experiments demonstrating primer-dependent transcription initiation by *Mtb* RNAP-σ^L holoenzyme on P-*sigL* derivatives having spacer ("sp") lengths of 4, 5, 6 and 7 bp (sequences in Supplementary Fig. 2; GpA-dependent transcription start in each case at template-strand position 3 nt upstream of nucleic-acid-scaffold dsDNA segment). Error bars, SE (N = 3).
Source data are provided as a Source Data file.

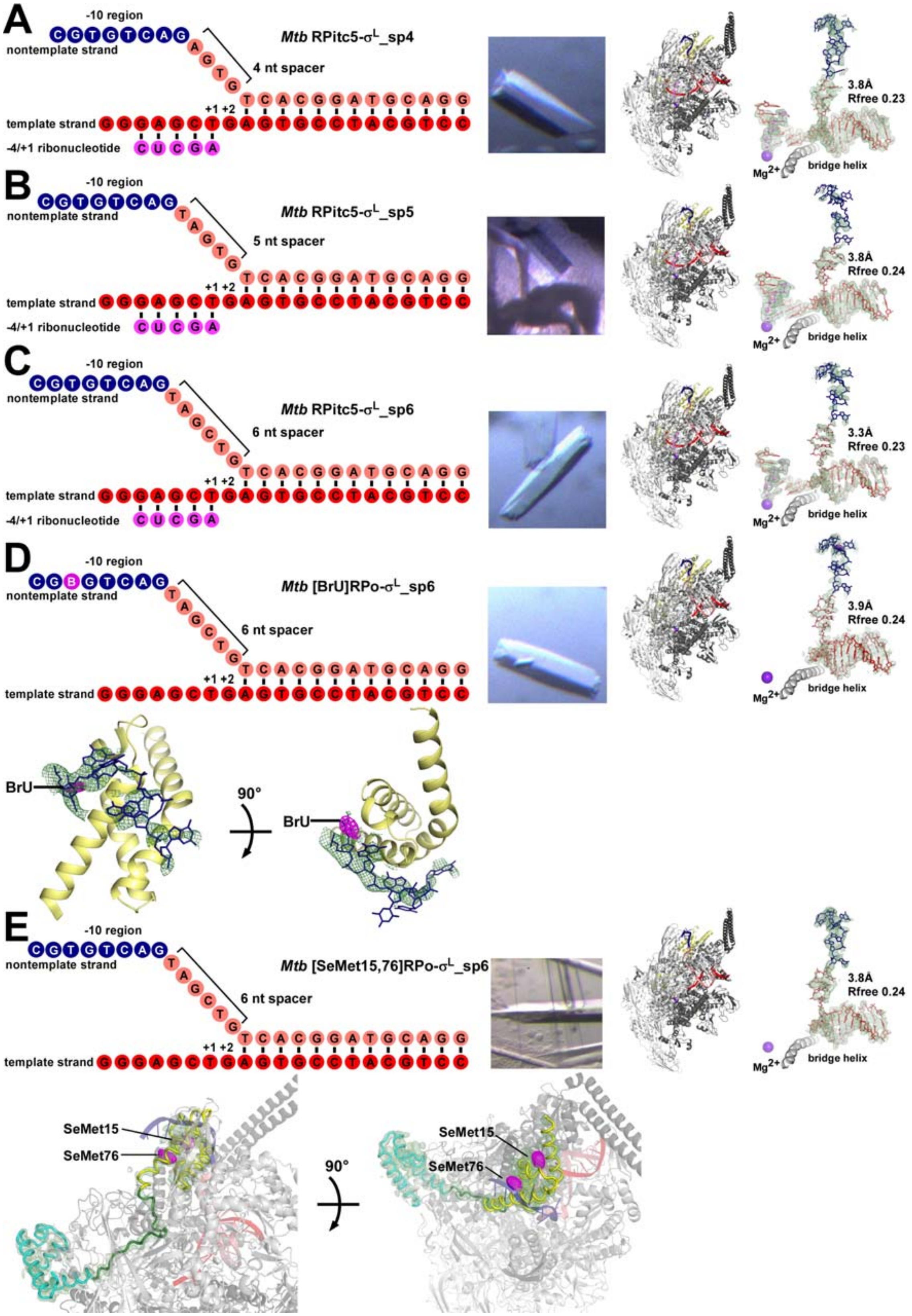

Supplementary Figure 2 (related to Fig. 1). Structure determination: nucleic-acid scaffolds,

crystals, and electron densities.

(A)-(C) Structure determination: *Mtb* -RPitc5-σ^L_sp4, *Mtb* RPitc5-σ^L_sp5, and *Mtb* RPitc5-σ^L_sp6. Left: nucleic-acid scaffold, colored as in Figs. 1-2 (sequences in blue designed based on -10 element of P-*sigL* promoter; sequences in pink and red designed based on nucleic-acid scaffold used for determination of structure of *Mtb* RPitc-σ^A in *1*). Center: crystal. Right: structure and experimental electron density. Green mesh, mF_o-DF_c electron-density omit map (contoured at 2.0σ).

(D) Structure determination: *Mtb* [BrU]RPo-σ^L_sp6 (analyzed to verify the translocational state of the transcription complex). Top: as in (A)-(C). Bottom: detail of electron density and Br anomalous difference density for promoter -10 element (two orthogonal views). Green mesh, mF_o-DF_c electron-density omit map (contoured at 2.0σ); magenta mesh, Br anomalous difference density (contoured at 3.0σ).

(E) Structure determination: *Mtb* [SeMet15,76]RPo-σ^L_sp6 (analyzed to verify the fit of σ^L). Top: as in (A)-(D). Bottom: detail of electron density and Se anomalous difference density for σ^L (two orthogonal views). Green mesh, mF_o-DF_c electron-density omit map (contoured at 2.0σ); magenta mesh, Se anomalous difference density (contoured at 3.0σ).

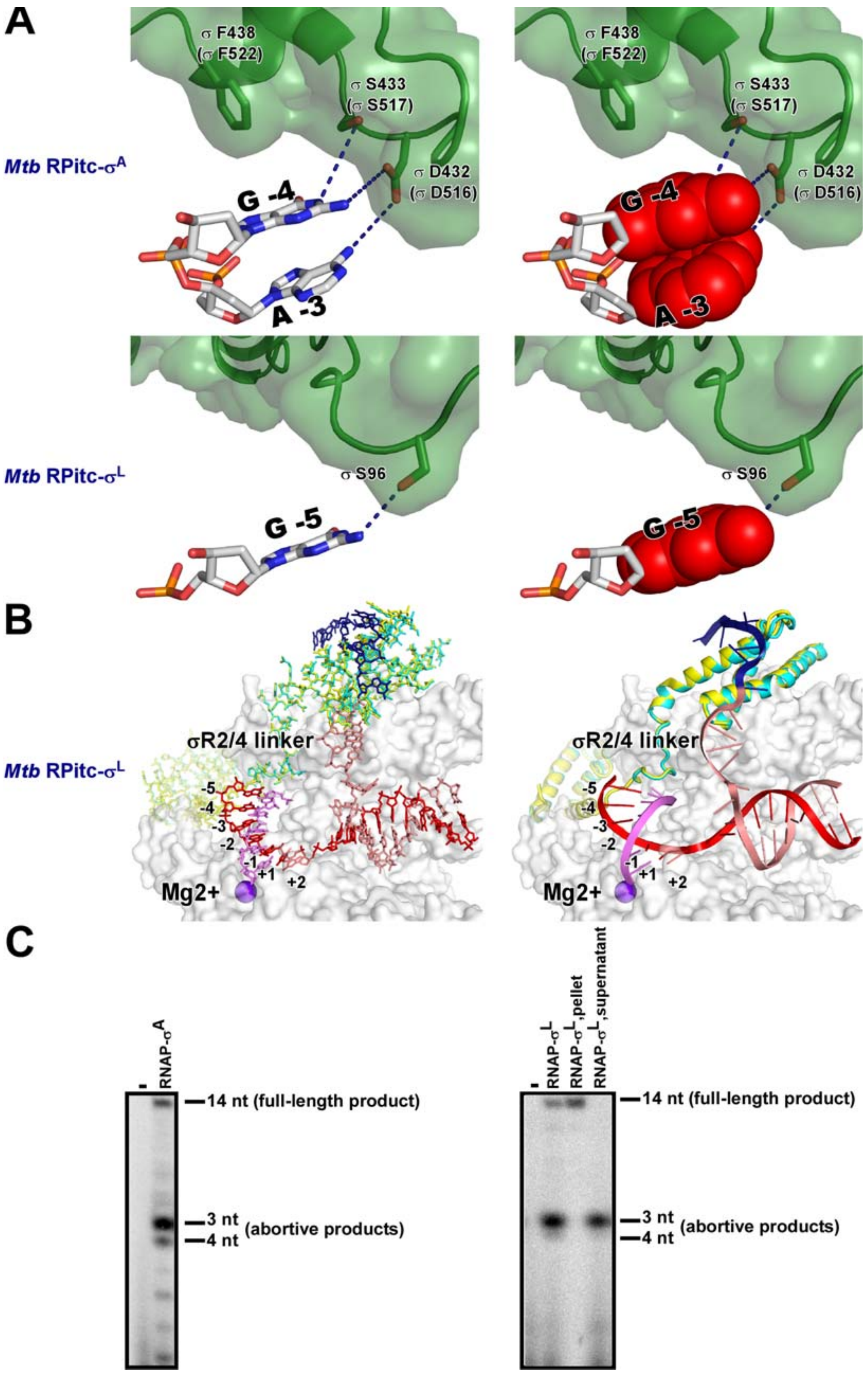

Supplementary Figure 3 (related to Figs. 3-4). Comparison of protein-nucleic acid interactions with

group-1 and ECF σ factors: interactions of σ^A σR3/4 linker and σ^L σR2/4 linker with transcription-bubble template-strand ssDNA

(A) Top: interactions of *Mtb* σ^A σR3/4 linker with template-strand nucleotides -4 and -3. Bottom: interactions of *Mtb* σ^L σR2/4 linker with template-strand nucleotide -5.Green surfaces, solvent-accessible surfaces of *Mtb* σ^L σR2/4 linker; red surfaces, van der Waals surfaces of template-strand base moieties; green ribbons, *Mtb* σ^L σR2/4 linker backbone; green and green-red stick representations, σ^L carbon and oxygen atoms, respectively; white, blue, red, and orange stick representations, DNA carbon, nitrogen, oxygen, and phosphorous atoms, respectively; blue dashed lines, H-bonds. Residues are numbered as in *Mtb* RNAP, σ^A, and σ^L, and, in parentheses, as in *E. coli* RNAP and σ^{70}.

(B) Superimposition of σ^L in RPitc (cyan; 5 nt RNA; *Mtb* RPitc5σ^L_sp6) on σ^L in RPo (yellow; 0 nt RNA; *Mtb* [SeMet15,76]RPo-σ^L_sp6). Left: all-atoms representation of σ^L; right: ribbon representation of σ^L. The observation that the conformation of the σ^L σR2/4 linker is identical in RPitc5 and RPo indicates that the 5'-end of RNA does not clash with the σ^L σR2/4 linker when RNA is ≤5 nt in length. Molecular modeling indicates that the 5'-end of RNA will clash with σ^L σR2/4 linker when RNA is >5 nt in length.

(C) Productive transcription initiation (14 nt RNA products) and abortive transcription initiation (3-4 nt RNA products) by *Mtb* RNAP-σ^A holoenzyme and *Mtb* RNAP-σ^L holoenzyme. Left panel and lanes 1-2 in right panel show results of transcription experiments; lanes 3-4 in right panel show results of transcript-release experiments (non-released products and released products in lane 3 and 4 , respectively). For both *Mtb* RNAP-σ^A holoenzyme and *Mtb* RNAP-σ^L holoenzyme, the principal abortive products with the analyzed initial transcribed sequence are 3 nt and 4 nt in length [ApApU and ApApUpU; identities confirmed by reference to products of parallel reactions omitting ATP and GTP; identities further confirmed by reference to products of parallel reactions with *E. coli* RNAP σ^{70} (see *5*)], consistent with previous results suggesting that abortive-product distributions are determined primarily by the initial transcribed sequence (*6*-*7*).

Source data are provided as a Source Data file.

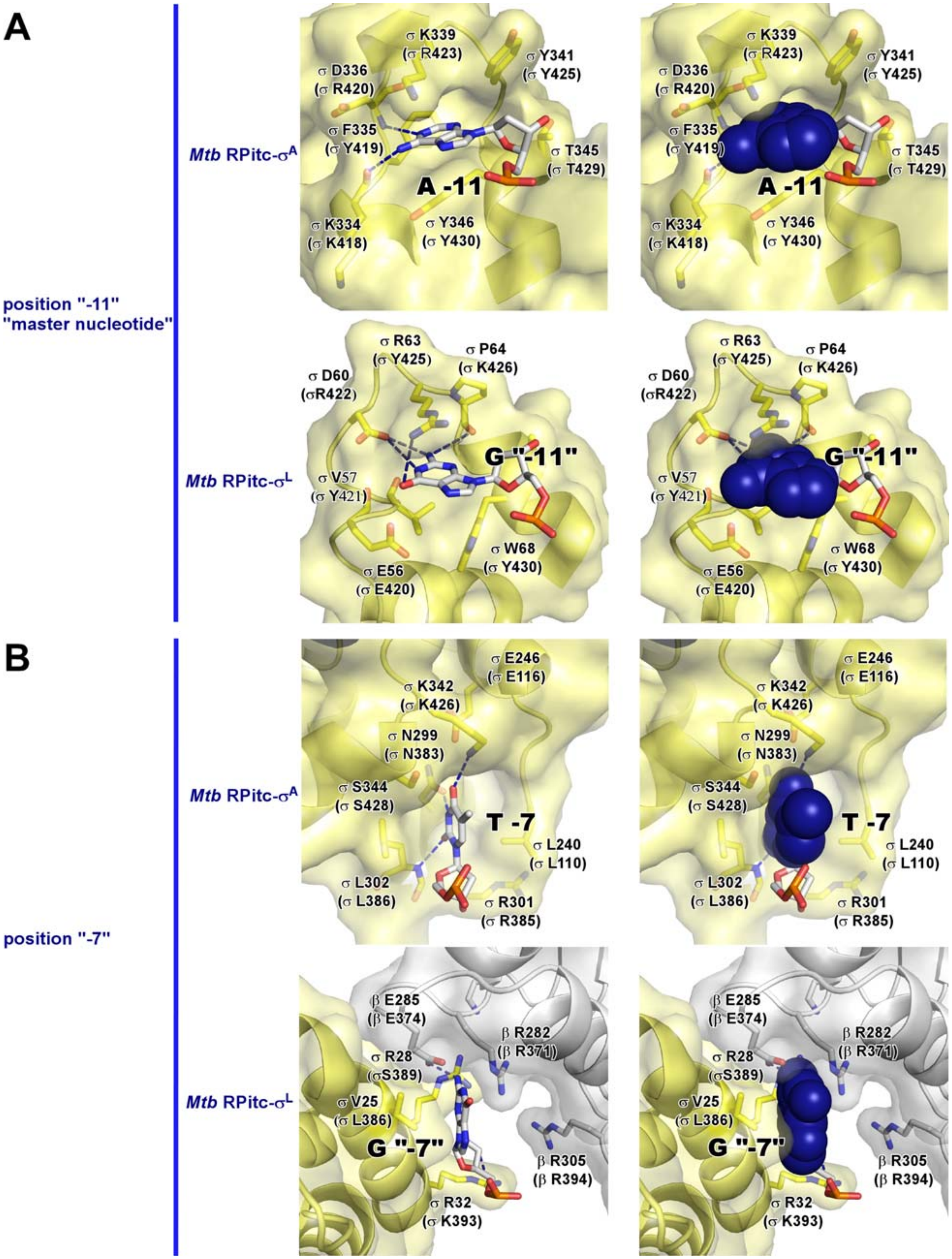

Supplementary Figure 4 (related to Figs. 3-5). Comparison of protein-nucleic acid interactions with

group-1 and ECF σ factors: interactions with unstacked, flipped nucleotides of promoter -10 element inserted into pockets of σR2

(A) Interactions of *Mtb* σ^A with "master nucleotide" position -11 (top) and *Mtb* σ^L with "master nucleotide" position "-11" (bottom).

(B) Interactions of *Mtb* σ^A with position -7 (top) and *Mtb* σ^L with position "-7" (bottom).

Colors are as in Fig. 5. Residues are numbered as in Supplementary Fig. 3.

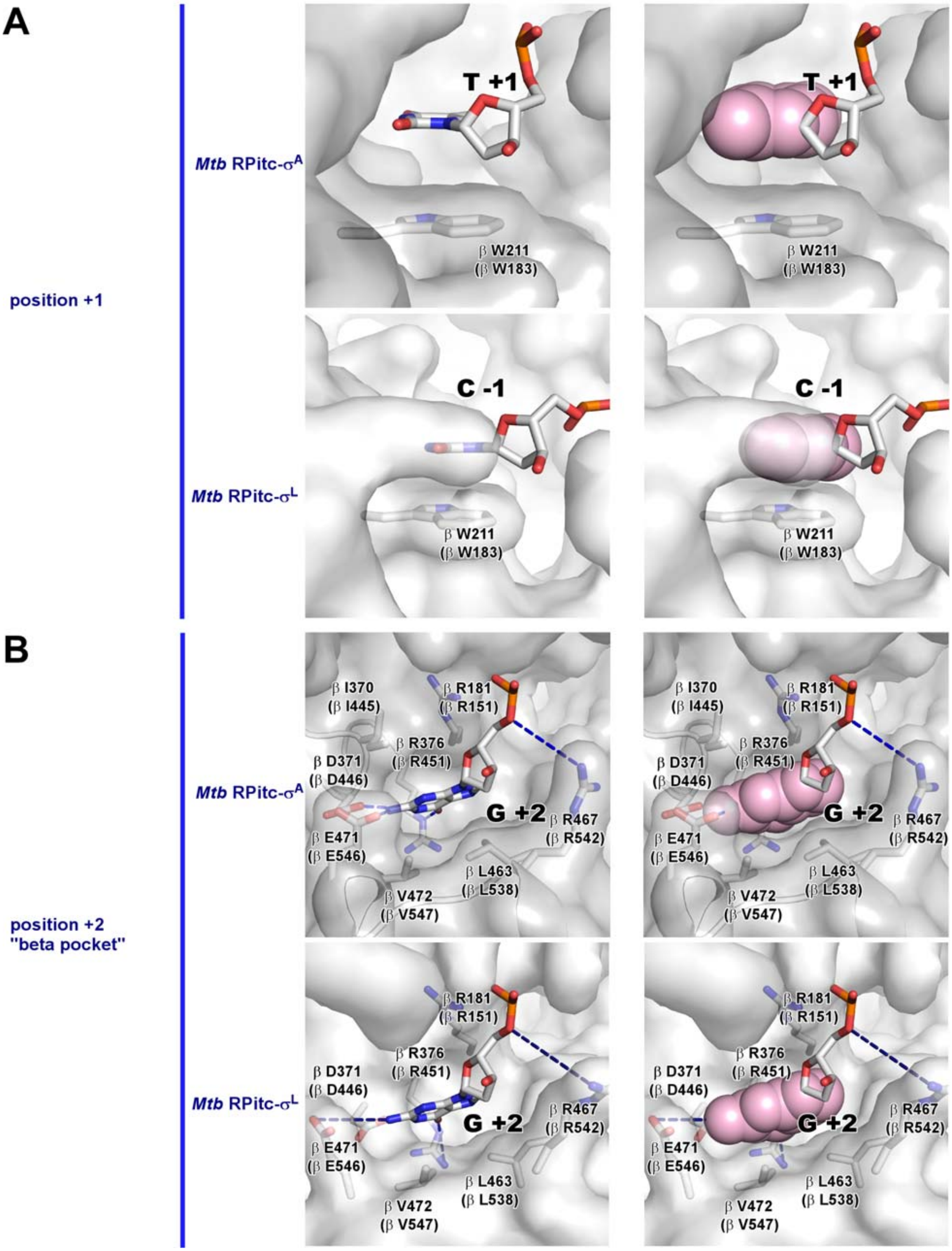

Supplementary Figure 5 (related to Figs. 3-4). Comparison of protein-nucleic acid interactions with

group-1 and ECF σ factors: interactions with promoter core recognition element (CRE).
(A) Stacking interactions of nontemplate-strand position +1 nucleotide on RNAP β subunit Trp211 in complexes of *Mtb* RPitc-σ^A (top) and *Mtb* RPitc-σ^L (bottom).
(B) Unstacking, flipping, and inserting of nontemplate-strand +2 nucleotide into pocket formed by RNAP β subunit ("beta pocket") in complexes of *Mtb* RPitc-σ^A (top) and *Mtb* RPitc-σ^L (bottom).
Colors are as in Fig. 5. Residues are numbered as in Supplementary Fig. 3.

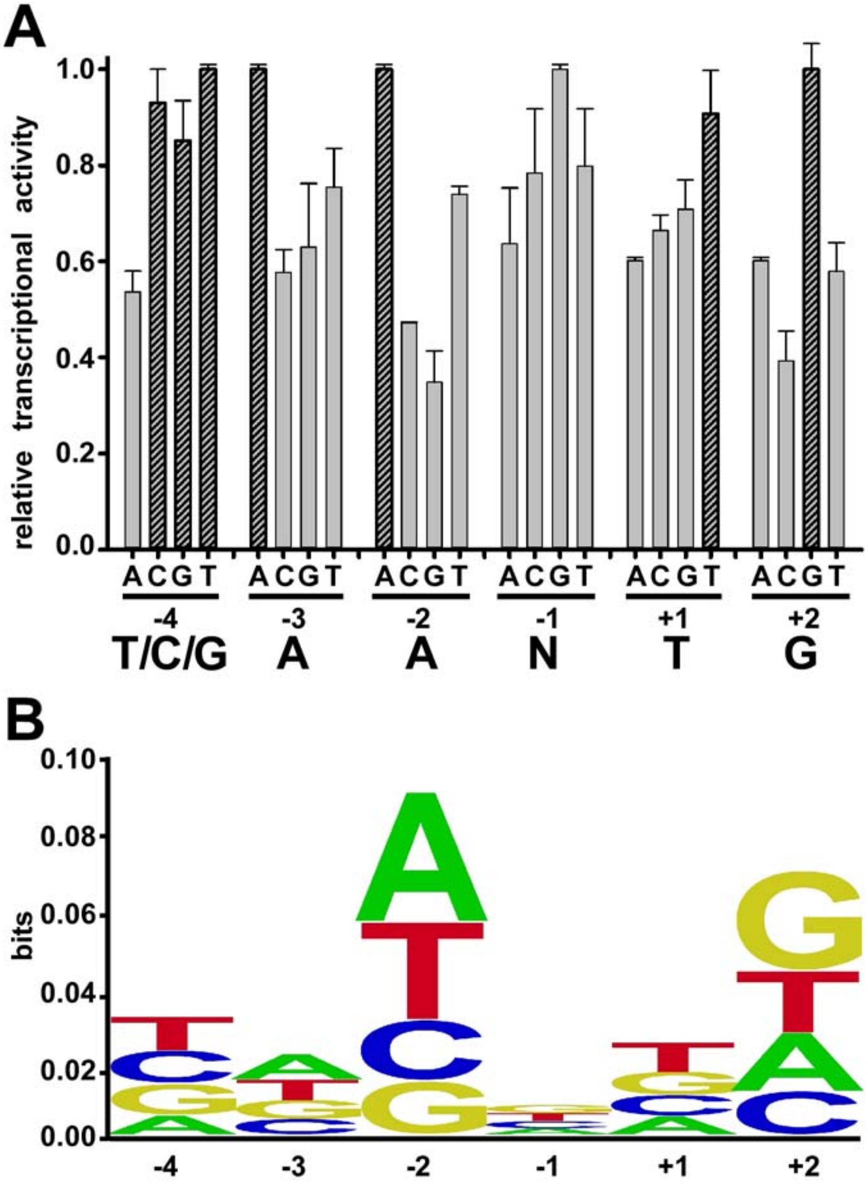

Supplementary Figure 6 (related to Fig. 6). Recognition by *Mtb* σ^L of σ^L-promoter core recognition element (CRE): experimental data

(A) Systematic-substitution experiments defining σ^L-promoter CRE consensus sequence. Relative transcription activities of derivatives of the σ^L-dependent promoter P-*sigL* having all possible single-base-pair substitutions at each position of CRE element (positions -4 through +2). Inferred consensus nucleotides are shown at bottom, and data for inferred consensus nucleotides are hatched. Error bars, SE (N = 3). **(B)** Sequence logo for σ^L-promoter CRE consensus sequence [generated using transcription data from (A) and enoLOGOS (*8*; http://biodev.hgen.pitt.edu/enologos/; input setting "energy (2)" and weight type setting "probabilities").

Source data are provided as a Source Data file.

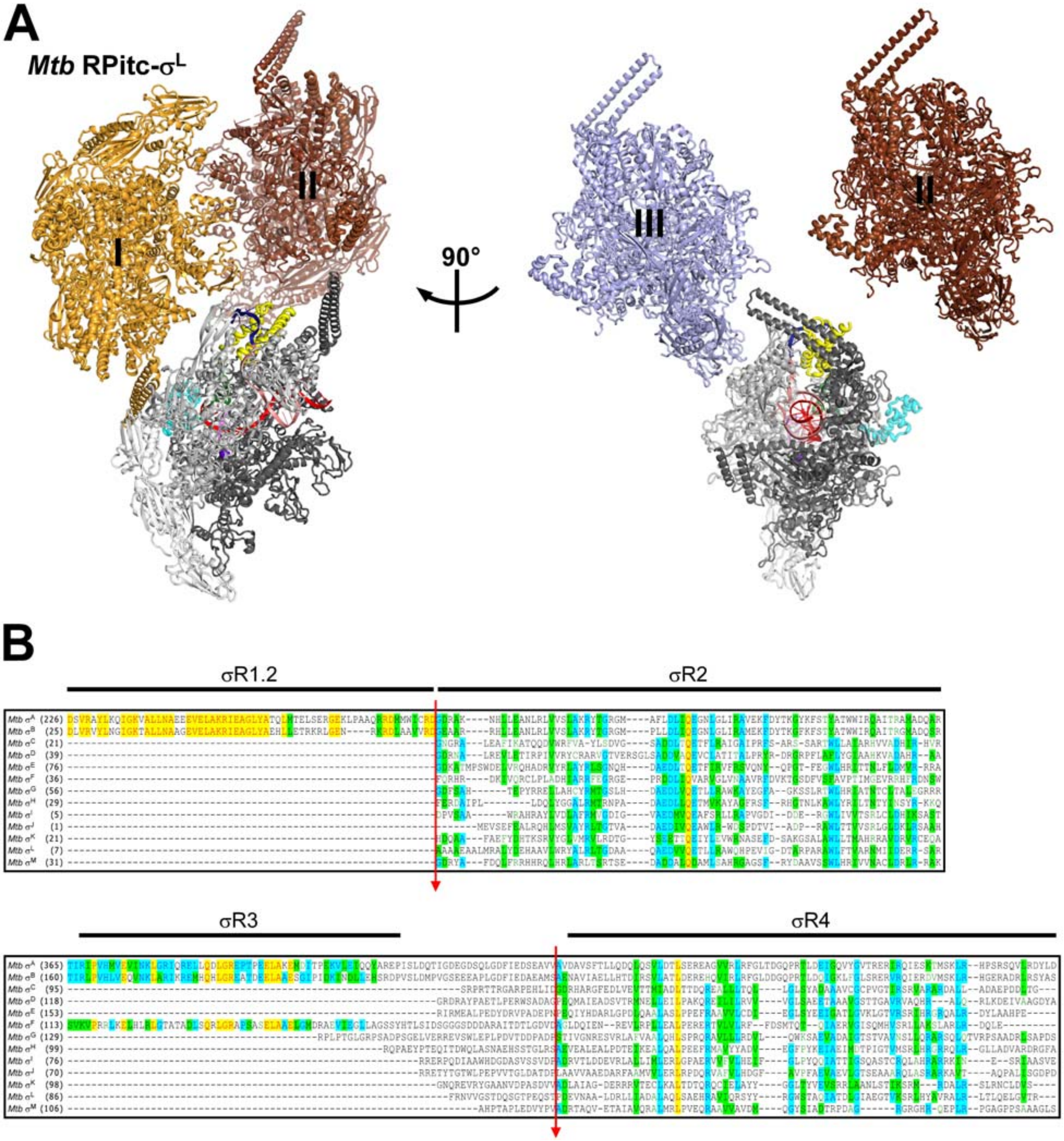

Supplementary Figure 7 (related to Figs. 1-4). Platform for systematic structural characterization of *Mtb* σ factors

(A) Crystal-lattice interactions in crystal form of this work. Figure shows *Mtb* RPitc-σ^L (colored as in Figs. 1-4) and the three crystal-lattice neighbors closest to σ^L in *Mtb* RPitc-σ^L (lattice neighbors I, II, and III in orange, rust, and violet, respectively). Two orthogonal view orientations are shown; for clarity, lattice neighbor III is omitted in first view orientation and lattice neighbor I is omitted in second view orientation. σ^L σR2 and σ^L σR2/4 linker of *Mtb* RPitc-σ^L make no interactions with lattice neighbors.

(B) Sequence alignment of the 13 *Mtb* σ factors: *Mtb* σ^A, σ^A, σ^B, σ^C, σ^D, σ^E, σ^F, σ^G, σ^H, σ^I, σ^J, σ^K, σ^L, and σ^M. Red arrows indicate proposed fusion sites for construction of chimeric σ factors comprising σR1.2-σR2 of *Mtb* σ^A through σ^M fused to σR2/4 linker-σR4 of *Mtb* σ^L (top red arrow) or comprising σR1.2-σR2/4 linker of *Mtb* σ^A-σ^M fused to σR4of *Mtb* σ^L (bottom red arrow).

SUPPLEMENTAL REFERENCES

1. Lin, W., Mandal, S., Degen, D., Liu, Y., Ebright, Y. W., Li, S., Feng, Y., Zhang, Y., Mandal, S., Jiang, Y., Liu, S., Gigliotti, M., Talaue, M., Connell, N., Das, K., Arnold, E. & Ebright, R. H. Structural basis of *Mycobacterium tuberculosis* transcription and transcription inhibition. *Mol Cell* 66, 169-179 (2017).

2. Hahn, M. Y., Raman, S., Anaya, M. & Husson, R. N. The Mycobacterium tuberculosis extracytoplasmic-function sigma factor SigL regulates polyketide synthases and secreted or membrane proteins and is required for virulence. *J. Bacteriol.* 187, 7062-7071 (2005).

3. Dainese, E., Rodrigue, S., Delogu, G., Provvedi, R., Laflamme, L., Brzezinski, R., Fadda, G., Smith, I., Gaudreau, L., Palù, G. & Manganelli, R. Posttranslational regulation of *Mycobacterium tuberculosis* extracytoplasmic-function sigma factor sigma L and roles in virulence and in global regulation of gene expression. *Infect. Immun.* 74, 2457-2461 (2006).

4. Rodrigue, S., Brodeur, J., Jacques, P., Gervais, A., Brzezinski, R. & Gaudreau, L. Identification of mycobacterial sigma factor binding sites by chromatin immunoprecipitation assays. *J. Bacteriol.* 189, 1505-1513 (2007).

5. Borowiec, J. & Gralla, J. Supercoiling response of the lac p^s promoter in vitro. *J. Mol. Biol.* 184, 587-598 (1985).

6. Hsu, L., Cobb, I., Ozmore, J., Khoo, M., Nahm, G., Xia, L., Bao, Y. & Ahn, C. Initial transcribed sequence mutations specifically affect promoter escape properties. *Biochemistry.* 45, 8841-8854 (2006).

7. Skancke, J., Bar, N., Kuiper, M. & Hsu, L. Sequence-dependent promoter escape efficiency is strongly influenced by bias for the pretranslocated state during initial transcription. *Biochemistry* 54, 4267-4275 (2015).

8. Workman, C., Yin, Y., Corcoran, D., Ideker, T., Stormo, G. & Benos, P. enoLOGOS: a versatile web tool for energy normalized sequence logos. Nucl. Acids Res. 33, W389-W392 (2005).
