# Supplementary material for: Structural basis of ECF-σ-factor-dependent transcription initiation
Source: Nat Commun. 2019 Feb 12;10:710. doi: 10.1038/s41467-019-08443-3 (PMC6372665; doi:10.1038/s41467-019-08443-3)
Supplement: Supplementary file 4 — Reporting Summary Checklist [file 41467_2019_8443_MOESM4_ESM.pdf]

nature research

Corresponding author(s): Richard H. Ebright

Reporting Summary

Nature Research wishes to improve the reproducibility of the work that we publish. This form provides structure for consistency and transparency in reporting. For further information on Nature Research policies, see Authors & Referees and the Editorial Policy Checklist.

Statistical parameters

When statistical analyses are reported, confirm that the following items are present in the relevant location (e.g. figure legend, table legend, main text, or Methods section).

n/a	Confirmed	
☐	☒	The exact sample size (*n*) for each experimental group/condition, given as a discrete number and unit of measurement
☐	☒	An indication of whether measurements were taken from distinct samples or whether the same sample was measured repeatedly
☒	☐	The statistical test(s) used AND whether they are one- or two-sided<br>*Only common tests should be described solely by name; describe more complex techniques in the Methods section.*
☒	☐	A description of all covariates tested
☒	☐	A description of any assumptions or corrections, such as tests of normality and adjustment for multiple comparisons
☒	☐	A full description of the statistics including central tendency (e.g. means) or other basic estimates (e.g. regression coefficient) AND variation (e.g. standard deviation) or associated estimates of uncertainty (e.g. confidence intervals)
☒	☐	For null hypothesis testing, the test statistic (e.g. *F*, *t*, *r*) with confidence intervals, effect sizes, degrees of freedom and *P* value noted<br>*Give P values as exact values whenever suitable.*
☒	☐	For Bayesian analysis, information on the choice of priors and Markov chain Monte Carlo settings
☒	☐	For hierarchical and complex designs, identification of the appropriate level for tests and full reporting of outcomes
☒	☐	Estimates of effect sizes (e.g. Cohen's *d*, Pearson's *r*), indicating how they were calculated
☐	☒	Clearly defined error bars<br>*State explicitly what error bars represent (e.g. SD, SE, CI)*

Our web collection on statistics for biologists may be useful.

Software and code

Policy information about availability of computer code

Data collection	The following computer software was used for data collection and reduction:<br>HKL2000 (Otwinowski et al., 1997)
Data analysis	The following computer software was used for data analysis:<br>MOLREP (Collaborative Computational Project, 1994)<br>Coot (Emsley et al., 2010)<br>Phenix (Adams et al., 2010)

For manuscripts utilizing custom algorithms or software that are central to the research but not yet described in published literature, software must be made available to editors/reviewers upon request. We strongly encourage code deposition in a community repository (e.g. GitHub). See the Nature Research guidelines for submitting code & software for further information.

Data

Policy information about availability of data

All manuscripts must include a data availability statement. This statement should provide the following information, where applicable:

- Accession codes, unique identifiers, or web links for publicly available datasets
- A list of figures that have associated raw data
- A description of any restrictions on data availability

Atomic coordinates and structure factors for the crystal structures of Mtb RPitc5-sL_sp4, RPitc5sL_sp5, RPitc5-sL_sp6, [BrU]RPo-sL_sp6, and [SeMet15,76]RPo-sL_sp6 have been deposited in the PDB with accession codes PDB 6DV9, 6DVB, 6DVC, 6DVD, and 6DVE.

Field-specific reporting

Please select the best fit for your research. If you are not sure, read the appropriate sections before making your selection.

☒ Life sciences ☐ Behavioural & social sciences ☐ Ecological, evolutionary & environmental sciences

For a reference copy of the document with all sections, see nature.com/authors/policies/ReportingSummary-flat.pdf

Life sciences study design

All studies must disclose on these points even when the disclosure is negative.

Sample size	Not relevant (no P-value testing).
Data exclusions	None.
Replication	Biochemical experiments were performed in triplicate. Results were consistent.
Randomization	Not relevant (no P-value testing).
Blinding	Not relevant (no P-value testing).

Reporting for specific materials, systems and methods

Materials & experimental systems

n/a	Involved in the study
☐	☒ Unique biological materials
☒	☐ Antibodies
☒	☐ Eukaryotic cell lines
☒	☐ Palaeontology
☒	☐ Animals and other organisms
☒	☐ Human research participants

Methods

n/a	Involved in the study
☒	☐ ChIP-seq
☒	☐ Flow cytometry
☒	☐ MRI-based neuroimaging

Unique biological materials

Policy information about availability of materials

Obtaining unique materials	Unique biological materials (bacterial strains and plasmids) developed as part of this work will be distributed upon request to qualified investigators.
